# UNDERSTANDING THE MEANING OF CARE: FROM THE PERSPECTIVE OF SPOUSE CAREGIVERS OF PEOPLE WITH PARKINSON’S DISEASE

**DOI:** 10.1093/geroni/igad104.2527

**Published:** 2023-12-21

**Authors:** JuHee Lee, Subin Yoo, Eunyoung Kim, Hyeran Park, Yun Joong Kim, Seok Jong Chung, Jee-Hye Yoo

**Affiliations:** Yonsei University, Seoul, Republic of Korea; Yonsei University, Seoul, Republic of Korea; Yonsei University, Seoul, Republic of Korea; Yonsei University, Seoul, Republic of Korea; Yonsei University College of Medicine, Seoul, Republic of Korea; Yonsei University College of Medicine, Seoul, Republic of Korea; CHA University, Pocheon, Republic of Korea

## Abstract

Parkinson’s disease is a highly prevalent geriatric disease in people aged 60 years and over. Parkinson’s disease can largely affect an individual’s life because it causes diverse motor and non-motor symptoms. As symptoms worsen, people with Parkinson’s disease are likely to become dependent, which can lead to caregiver burden. Therefore, the aim of this study is to understand the meaning of care in spouse caregivers who care for people with Parkinson’s disease. This study is a qualitative study using face-to-face in-depth interviews. A total of 15 spouse caregivers (10 husbands and 5 wives) were recruited from a neurological outpatient clinic at a tertiary hospital in South Korea. Two trained nurses interviewed spousal caregivers, and thematic analysis was used for data analysis. In this study, four themes were identified: (a) symptom management, (b) struggling to care, (c) intimacy with one’s spouse, and (d) uncertainty of the future illness progress. People with Parkinson’s disease experienced at least more than one motor or non-motor symptoms. Physical function and lifestyle modification of people with Parkinson’s disease, caregivers’ positive attitude towards the care role, and intimate relationships were key factors of self-care. For caregivers, the progression of disease was the major concern of care. Given that the number of older households is increasing in Asia, it is important to understand older spouse caregivers’ perspective for care of people with Parkinson’s disease. By understanding their needs and difficulties, healthcare providers can build further plans for them.

